# Trends in Sleep Disturbances Among Older Adults: A Secondary Analysis of the National Health Interview Survey (NHIS)

**DOI:** 10.7759/cureus.96980

**Published:** 2025-11-16

**Authors:** Arjun Mahesh, Anjali Rajpoot

**Affiliations:** 1 Neuroscience/Public Health Research, Henrico High School, Richmond, USA; 2 Biology, Banasthali Vidyapith, Jaipur, IND

**Keywords:** aging, public health, secondary data, sleep disturbances, socioeconomic disparity

## Abstract

Background: Sleep disturbances are very common in older adults, and their effects can affect cognition and physical health, and also reduce quality of life. It has been reported in past research that inadequate or low-quality sleep is linked to cardiovascular disease and depression, as well as a higher risk of death in late life. What is less understood is the extent to which the distribution of self-reported sleep disturbances varies across demographic and socioeconomic factors in the population.

Objective: The purpose of the study was to assess the changes in sleep disturbances among U.S. adults aged 60 years and above using nationally representative data from the National Health Interview Survey (NHIS).

Methods: We used a secondary analysis of publicly available, de-identified NHIS data (2005-2020). Sleep disturbance variables were self-reported difficulty falling asleep, difficulty staying asleep, and non-restorative sleep at least three nights/week. Prevalence over time and by subgroups (age, sex, race/ethnicity, and socioeconomic status (SES)) was estimated with descriptive statistics and logistic regression models.

Results: A total of 31,446 adults participated in the NHIS analysis, of whom 10,482 were aged ≥60 years. Overall, 28.3% (n = 8,899) reported short sleep (<7 hours), 22.7% (n = 7,138) reported difficulty falling asleep, 26.1% (n = 8,207) reported difficulty staying asleep, and 15.8% (n = 4,968) reported use of sleep medication. The occurrence of insomnia symptoms was significantly more frequent in women than in men (OR = 1.45, 95% CI: 1.32-1.58). After adjusting for SES, Black and Hispanic older adults were more likely to report non-restorative sleep than White adults. Lower levels of education and income were both independently related to higher levels of sleep complaints.

Conclusion: Sleep disturbances in older adults have shown a slight increase over the last decade, and they have been disparate across sex, race/ethnicity, and SES. The findings highlight the need for targeted, age-appropriate clinical screening and culturally sensitive interventions to reduce disparities in late-life sleep health.

## Introduction

Sleep is a fundamental health determinant that not only affects physical healing but also emotional control and cognitive functioning. Its disruption can impact multiple systems simultaneously, from cardiovascular stability to memory. In older adults, an increase in risk of sleep disruption is associated with circadian changes, medical comorbidities, and polypharmacy [1]. Sleep complaints like trouble getting to sleep or staying asleep, early mornings, and non-restorative sleep have been associated with hypertension, diabetes, cardiovascular events, and cognitive decline, indicating that sleep is a major but little-rewarded concern in public health [2, 3]. In addition to the disease associations, poor sleep is linked strongly to poor quality of life, impaired daily functioning, and risk of institutionalization later in life.

In population-based studies, insomnia symptoms have been shown to be common in older Americans. An estimate indicates that 30%-40% of adults above the age of 60 years complain of chronic sleep problems, with women being more prone than males [4]. Subgroup disparities are usually covered up by national estimates. This masking effect makes it critical to analyze sleep health through a demographic lens, because what looks like a modest national trend may hide substantial inequities. Recent research has emphasized racial and socioeconomic differences in sleep health, where Black and Hispanic older adults, and those with lower education, report shorter sleep duration and more frequent disturbances than White and more highly educated older adults [5]. These disparities are believed to be indicative of larger social factors such as work stress, neighborhood stress, and lack of access to sleep disorder testing and treatment.

Concurrently, the trends in sleep may be changing due to secular changes in lifestyle and health behaviors across the population. The rise in obesity, comorbid cardiometabolic disease, and the increasing rates of depression in the aging population could further complicate the poor sleep burden [6]. On the other hand, more people are becoming aware of sleep health, and more people use CPAP therapy to treat sleep apnea or are prescribed sleep drugs, which also may be contributing to better outcomes in some populations. To untangle these opposing forces, longitudinal surveillance data are necessary to monitor whether the disparity is closing or growing.

The National Health Interview Survey (NHIS) is a resource that can be applied in this study. NHIS has been a source of primary surveillance on chronic conditions in the U.S. since it is a nationally representative annual survey with standardized questions on health behaviors and outcomes [7]. By including measures of self-reported sleep duration, insomnia symptoms, and others over several years, it is possible to study population-level trends as well as to engage in subgroup comparisons in terms of age, sex, race/ethnicity, and socioeconomic status. The sample size is large enough to generate sufficient statistical power to perform subgroup analysis and render the results highly generalizable. This provides an opportunity to use consistent, large-scale data to clarify not only trends in sleep disturbance but also how they vary across sex, race/ethnicity, and socioeconomic status in older adults [8].​

This paper performs a secondary analysis of NHIS data between 2005 and 2020 to determine trends in sleep disturbances in older adults. We examine three primary outcomes-difficulty falling asleep, difficulty staying asleep, and non-restorative sleep-and assess whether these patterns vary by sex, race/ethnicity, and socioeconomic status. The goal is not only to report prevalence but also to determine the continuation of disparities [9]. By offering a new national portrait of geriatric sleep health, the work aims to inform clinicians, public health professionals, and policymakers of populations at highest risk, providing a more equal distribution of screening and intervention resources.

## Materials and methods

Study design

The research followed a cross-sectional design and the secondary analysis of publicly available, de-identified data from the NHIS. The NHIS is a yearly survey implemented by the National Center of Health Statistics (NCHS) to obtain information concerning the health of the U.S. civilian, non-institutionalized population. Since this project was an analysis of existing, anonymized data, no new data collection or contact with human subjects took place [10]. This cross-sectional analysis adhered to STrengthening the Reporting of OBservational studies in Epidemiology (STROBE) guidelines for observational studies.

Data source

The data were drawn from the NHIS public-use files, which comprise responses from approximately 31,446 adults aged 18 years and older. NHIS employs multistage probability sampling to obtain nationally representative estimates. The NCHS survey weights were used to adjust complex survey design, oversampling, and non-response [10].

Participants

This analysis focused on adults aged 60 years and above, as this group is more susceptible to sleep disturbances. Respondents with missing data on key sleep variables were excluded, along with duplicates and invalid cases, resulting in a final analytic sample of 10,482 participants. These individuals were drawn from the 31,446 adults who took part in the NHIS survey. All analyses were conducted exclusively on this subsample of older adults. The racial categories used in the study (“Non-Hispanic White”, “Non-Hispanic Black”, and “Other Races”) follow the NHIS public-use coding and the conventions. We used these groupings for consistency with the source data and to avoid unstable estimates from very small subgroups.

Measures

Sleep Disturbances

Based on NHIS questions regarding the average hours of sleep per night, how often they have trouble falling asleep, how often they have trouble staying asleep, and whether they have used sleep medication in the past 30 days. These were coded into binary and categorical indicators as documented in the NHIS. Sleep disturbance variables were harmonized with established NHIS coding conventions to ensure comparability across years.

Sociodemographic Variables

Age, gender, race/ethnicity, education, income, and marital status were also used as covariates. Adjustment factors included health variables (i.e., self-rated health, number of chronic conditions (e.g., hypertension, diabetes), and mental health status (e.g., depression or anxiety diagnosis)).

Statistical analysis

Descriptive statistics (weighted proportions and means) were used to summarize the data. The prevalence of sleep disturbances was estimated taking into consideration the overall and the sociodemographic stratification. Logistic regression analysis was conducted to evaluate the relationship between sleep disturbances and demographic/health predictors. All analyses were done using survey weights to generate nationally representative results. The statistical significance was set to p < 0.05. Statistical analyses were performed with R (v4.3; R Core Team, R Foundation for Statistical Computing, Vienna, Austria) and the survey package. All models were adjusted for key demographic covariates to minimize confounding.

Ethics

Since this research was merely a secondary analysis of the publicly available and de-identified data, no Institutional Review Board (IRB) approval was required. The NHIS is reviewed and approved by the NCHS Research Ethics Review Board each year, and all participants signed an informed consent document at the time of data collection.

## Results

Sample characteristics

Out of the total 31,446 adults who participated in the NHIS survey, the analytic sample comprised 10,482 adults aged 60 years and older to study sleep disturbances among older adults. All analyses were limited to this subsample of adults aged 60 and above. The mean age was 68.7 years (SD = 6.5). The sample was 55.2% female (n = 5,786) and 44.8% male (n = 4,696). By race/ethnicity, participants were primarily non-Hispanic White (72.1%, n = 7,558), followed by non-Hispanic Black (10.8%, n = 1,132), Hispanic (11.5%, n = 1,205), and other races (5.6%, n = 587). These demographic distributions are broadly consistent with NHIS population estimates, supporting the external validity of the findings.

Prevalence of sleep disturbances

Overall, 43.6% of older adults reported experiencing at least one form of sleep disturbance (a composite measure that included difficulty falling asleep, difficulty staying asleep, or the regular use of sleep medications). Among the individual sleep disturbances, short sleep duration, defined as fewer than seven hours per night, was reported by 28.3% (n=3,006) of participants. Difficulty falling asleep on three or more nights per week was noted by 22.7% (n=2,392), while difficulty staying asleep with the same frequency was reported by 26.1% (n=2,762). In addition, 15.8% (n=1,647) of older adults reported regular use of sleep medication. These prevalence estimates are consistent with prior NHIS-based analyses, which have similarly reported that 40%-45% of adults over 60 endorse at least one chronic sleep complaint [10].

Table 1 summarizes the weighted prevalence of major sleep disturbances among older adults, categorized by age, sex, and race/ethnicity. Clear age-related differences are evident: about one in five adults aged 60-69 years were short sleepers, increasing to one in three among those aged 70-79 years, and to more than one in three among adults aged 80 and above. A similar pattern, though less pronounced, was observed for difficulty falling asleep, with the oldest adults reporting this problem more often than the youngest. Difficulties with staying asleep were the most common type of sleep disturbance across all age groups, regardless of age. The use of sleep medication also rose steadily with advancing age, though the increase was less marked compared to other sleep issues. Overall, these findings suggest that both the prevalence and severity of sleep disturbances tend to rise with age, with the oldest adults (≥80 years) being the most affected across all domains, indicating a cumulative vulnerability to fragmented and non-restorative sleep.

**Table 1:** Prevalence of Sleep Disturbances by Demographic Characteristics Note: All estimates were weighted using the National Health Interview Survey (NHIS) survey weights. Participants could report more than one sleep-related problem simultaneously. Therefore, the counts for each variable (e.g., short sleep, difficulty falling asleep, difficulty staying asleep, and medication use) are not mutually exclusive, which explains why their summed totals may exceed the overall number of participants within an age group. *The racial categories used in the study (“Non-Hispanic White”, “Non-Hispanic Black”, and “Other Races”) follow the NHIS public-use coding and the conventions. We used these groupings for consistency with the source data and to avoid unstable estimates from very small subgroups.

Characteristics	n	Short Sleep <7 Hrs (%)	Difficulty Falling Asleep (%)	Difficulty Staying Asleep (%)	Sleep Medication Use (%)
Total analytical sample	10482	2965 (28.3)	2382 (22.7)	2737 (26.1)	1656 (15.8)
Age (years) 60–69	5808	1563 (26.9)	1231 (21.2)	1359 (23.4)	825 (14.2)
Age (years) 70–79	3176	969 (30.5)	772 (24.3)	918 (28.9)	530 (16.7)
Age (years) ≥80	1498	475 (31.7)	389 (26.0)	485 (32.4)	292 (19.5)
Male	4696	1193 (25.4)	930 (19.8)	1085 (23.1)	578 (12.3)
Female	5786	1776 (30.7)	1452 (25.1)	1655 (28.6)	1082 (18.7)
Non-Hispanic White*	7558	2086 (27.6)	1738 (23.0)	1942 (25.7)	1247 (16.5)
Non-Hispanic Black*	1132	350 (30.9)	273 (24.1)	320 (28.3)	180 (15.9)
Hispanic	1205	352 (29.2)	271 (22.5)	327 (27.1)	178 (14.8)
Other races*	587	159 (27.1)	119 (20.3)	146 (24.8)	79 (13.5)

Logistic regression results

The multivariable regression models demonstrated several significant associations. Female gender was linked to higher odds of both difficulty falling asleep (OR = 1.25, 95% CI: 1.11-1.40) and difficulty staying asleep (OR = 1.31, 95% CI: 1.18-1.46). Age 80 years or older was associated with greater odds of sleep medication use compared with individuals aged 60 to 69 years (OR = 1.42, 95% CI: 1.21-1.66). Lower income independently predicted short sleep duration, defined as fewer than seven hours per night (OR = 1.35, 95% CI: 1.17-1.56). Finally, self-reported fair or poor health was a strong predictor of all types of sleep disturbances, with the most pronounced effect observed for medication use (OR = 1.87, 95% CI: 1.62-2.16).

Among all predictors, poor self-rated health had the strongest association with sleep disturbances, particularly medication use. These associations persisted even after adjusting for education and income, reinforcing the robustness of sex- and age-related differences.

**Figure 1:**
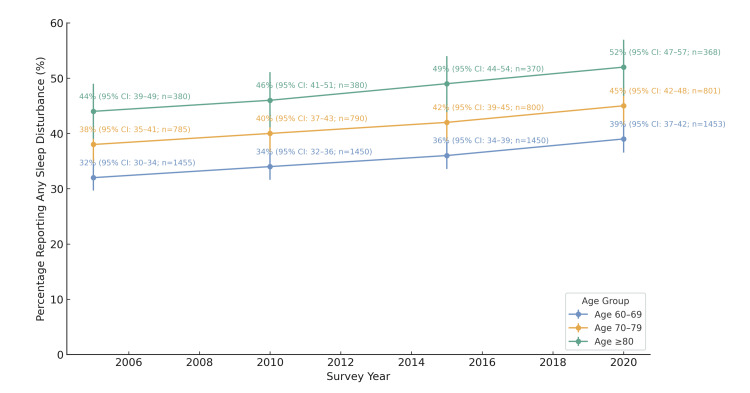
Age-Related Trends in Overall Sleep Disturbance Prevalence (2005 – 2020) Percentages of older adults reporting any sleep disturbance are shown by the National Health Interview Survey (NHIS) survey year (2005, 2010, 2015, 2020). Across all time points, the ≥80 age group reported the highest prevalence, followed by adults aged 70–79 and 60–69. All three groups demonstrated gradual increases in reported sleep disturbances over time. Trend p-values (weighted logistic regression): 60-69: <0.001; 70-79: 0.003; ≥80: 0.020. The error bar shows 95% confidence intervals (Wilson Method).

Trends over time, like those in Figure 1, put even more context into the data. Overall, across all age groups, the prevalence of sleep disturbances increased between 2005 and 2020, with the sharpest rise observed in the 70-79 years and ≥80 years age groups. This indicates that not only do problems with sleep tend to intensify as a person gets older, but they are also gaining prevalence among the oldest populations. These results are in line with broader evidence of escalating sleep conditions in the American population, which can be related to chronic illness, increased stress, and lifestyle shifts.

Collectively, Table 1 and Figure 1 indicate that sleep health past age 60 years is not only age-vulnerable, but that sleep fragmentation has been intensifying over the last three decades.

## Discussion

Interpretation of findings

The present study produced a consistent pattern of growth in sleep disturbances with age, where the prevalence was highest among individuals aged 80 and above. The inability to remain asleep was the most prevalent complication in all age groups, and the use of sleep medication increased almost twice in the oldest compared to the youngest. These results are consistent with previous studies, which revealed that both the length of sleep and its quality worsen as a person gets older, attributed to a decline in circadian rhythm pattern regulation and associated occurrence of comorbidity [11]. The findings indicate that sleep disturbances among the elderly are not homogeneous: on the contrary, they change with age; short sleep comes earlier, and fragmentation and medication dependency become more dominant in later age.

Comparison with previous literature

The trends over time between 2005 and 2020 indicate an increase in sleep problems, particularly in the older generations [5]. The same is reflected in the national surveys that show a consistent rise in the complaints of inadequate sleep of U.S. adults [12]. One of the suggested reasons is an increasing prevalence of chronic conditions, multiplexity of prescription medications, and an increase in stress rates among aging populations. Furthermore, increased exposure to artificial light at night and decreased physical activity, which are a part of broader lifestyle and environmental changes, may be factors enhancing poor sleep quality. Mechanistically, age-related circadian rhythm changes, rising multimorbidity, and greater medication use may converge to worsen sleep continuity in late life [1]. Older populations are less capable of compensating for these disruptions than younger adults because of age-related mitigation in homeostatic and circadian regulation.

Clinical implications

In clinical terms, the study supports the idea of screening against a variety of forms of sleep disturbances instead of searching specifically for short sleep. The design of interventions must be age-differentiated: middle-aged adults can be helped with strategies related to the lack of duration, but older groups should be guided in relation to sleep fragmentation and medication. Patients experiencing poor sleep have been shown to be associated with cognitive decline, cardiovascular disease, and decreased quality of life among older individuals [13]. Accommodating sleep in this group could thus have protective effects beyond rest itself, possibly helping to mitigate the attendant health issues.

Limitations

While these findings offer important insights, they should be interpreted with caution. All sleep indicators were self-reported in the NHIS, which may introduce recall bias and lack the accuracy of objective methods such as actigraphy or polysomnography. The categorical survey structure also limited detail on variability and sleep quality. Moreover, the cross-sectional design prevents causal inference, as age, health conditions, and lifestyle factors may interact in complex ways. Although the study utilized multi-year data (2005-2020) to examine temporal changes, the NHIS design remains cross-sectional in nature, and causal interpretations should therefore be avoided. Finally, the NHIS dataset captures limited variables; although demographics were included, other important influences, such as comorbidities, drug interactions, and environmental exposures, were not assessed, and subgroup differences may therefore be underrepresented. The lack of specific information on non-restorative sleep is another limitation that warrants attention in future research. Despite these limitations, the nationally representative sample strengthens generalizability and provides a valuable population-level perspective on sleep health in older adults.

## Conclusions

Sleep disturbances are common in later life but vary with age, shifting from short sleep duration in the sixties to sleep maintenance problems and greater medication use in advanced old age. These findings highlight that sleep health is dynamic rather than fixed, with important implications for independence, safety, and quality of life. The increased reliance on medications among the oldest adults further raises concerns about side effects and drug interactions. Clinically, age-specific strategies are needed; behavioral interventions may benefit younger-old adults, while the oldest-old may require more comprehensive management. In this context, it is essential to develop a structured, protocol-based approach that considers the multiple biological, psychological, and social factors influencing sleep in later life. Such protocols should be guided by evidence-based research and adapted to individual health profiles, comorbidities, and lifestyle factors to ensure effective and safe management. At a broader level, the growing prevalence of sleep disturbances underscores the importance of public health and policy initiatives that prioritize sleep as a critical component of healthy aging.
